# Barriers and enablers in the implementation of a program to reduce cesarean deliveries

**DOI:** 10.1186/s12978-017-0369-3

**Published:** 2017-08-29

**Authors:** Clara Bermúdez-Tamayo, Emilia Fernández Ruiz, Guadalupe Pastor Moreno, Gracia Maroto-Navarro, Leticia Garcia-Mochon, Francisco Jose Perez-Ramos, Africa Caño-Aguilar, Maria del Pilar Velez

**Affiliations:** 1 Andalusian School of Public Health, Cuesta del Observatorio 4 s/n, 18010 Granada, Spain; 2 CIBERESP, Ciber de Epidemiologia y Salud Publica, Madrid, Spain; 3 Asociación de Mujeres Politólogas, Granada, Granada, Spain; 4 Consejería de Igualdad, Salud y Políticas Sociales, Junta de Andalucía, Sevilla, Avd. De Hytasa n° 14, 41006 Sevilla, Spain; 5 UGC Obstetrics and Gynaecology Hospital Universitario San Cecilio, Av Doctor Oloriz, 16, 18012 Granada, Spain; 6 Department of Obstetrics and Gynecology, Kingston General Hospital, Queen’s University, Kingston, Canada

**Keywords:** Cesarean section, Clinical practice guidelines, Evidence-based medicine

## Abstract

**Background:**

Conducting audits, implementing best practices and giving feedback to the professionals have shown considerable promise in reducing rates of cesarean delivery and mother-child morbidity. The purpose of the study is two-fold: a) to identify the factors that facilitate change in current practices and thus reduce the use of obstetric interventions, and b) to better understand the barriers to such changes. To reach these objectives, the study analyzed the experiences of professionals participating in a program to reduce cesarean rates in 20 hospitals in Andalusia (Spain).

**Methods:**

A qualitative exploratory study was conducted. Participants were 14 ob-gyns and 14 nurse-midwives who work for Spain’s National Healthcare System and have been involved in the program. To gather information, in-depth individual interviews were used. The interview was designed to examine factors affecting the quality of care, such as issues related to policy/management, hospitals, practitioners and patients.

**Results:**

The barriers identified include: 1) At the policy/management level: limited influence of institutional policy and the scant political commitment perceived. 2) At the organizational level: separation of the hierarchical structure of doctors from that of nurse-midwives, few positive incentives and the strong threat of sanctions for malpractice, inappropriate reorganization of midwife/obgyns competences. 3) At the healthcare staff and facility level: reluctance to change accentuated by years of professional practice. 4) Physical resources: obsolete delivery rooms with a medical look. 5) At the professional level: medical and legal pressure, cesarean delivery considered safe in the event of a legal claim, low motivation due to decline in working conditions, convenience-based practices. 6) Woman giving birth and her family: fear of pain, impatience while waiting for process to occur, misinformation. The enablers include: 1) At the organizational level: good coordination with pediatrics and emergency departments, 2) Training: skills updates for a less-interventionist approach, increased awareness, 3) Health professionals: satisfaction for a job well done, recognition by patients. 4) Woman giving birth: information circuits for patients and their families, trust in health professionals.

**Conclusions:**

The results can contribute to the design of more effective knowledge translation interventions to reduce cesarean sections, based on overcoming obstacles, reinforcing enabling factors and attempting to (re)define the boundaries between research and practice.

## Plain English summary

High rates of cesarean deliveries constitute an important public health problem. Audits, implementing best practices and giving feedback to the professionals have shown considerable promise in reducing rates of cesarean deliveries and mother-child morbidity. This study aims to identify the factors that facilitate change in practice and thus reduce the use of obstetric interventions. We analyzed the experiences of ob-gyns and nurse-midwives who work in Spain’s National Healthcare System and have been involved in the program. The interviews examined factors related to policy/management, hospitals, practitioners and patients.

The barriers identified were: 1) At the management level: limited influence of institutional policy and the scant political commitment perceived. 2) At the organizational level: separation of the hierarchical structure of doctors from that of nurse-midwives, few positive incentives and the strong threat of sanctions for malpractice, inappropriate reorganization of midwife/ob-gyns competencies, 3) Healthcare staff and facility level: reluctance to change accentuated by years of professional practice. 4) Physical resources: obsolete delivery rooms with a medical view, 5) At the professional level: medical and legal pressure, cesarean deliveries considered safe in the event of a legal claim, low motivation due to decline in working conditions, convenience-based practices. 6) Woman giving birth and her family: fear of pain, impatience while waiting for process to occur, misinformation. The enablers include: 1) At the organizational level: good coordination with paediatrics and emergency departments, 2) Training: skills updates for a less-interventionist approach, increased awareness, 3) Health professionals: satisfaction for a job well done, recognition by patients. 4) Woman giving birth: information circuits for patients and their families, trust in health professionals.

These results allow a better understanding of the problems that remain latent and that emerge in the day-to-day activity of the healthcare professionals attending births in public hospitals.

## Background

High rates of cesarean deliveries constitute an important public health problem, not only because of the potential maternal and perinatal complications resulting from interventions without clinical justification, but also because of long-term complications and higher associated costs [1].

According to the World Health Organization, at the population level, caesarean section (CS) rates higher than 10% are not associated with reductions in maternal and newborn mortality rates [1]. In 2015, the rate of cesarean deliveries in Spain was 25.3% [2], considerably higher than the figure deemed acceptable [3]. In the region of Andalusia, the rates range from 16% to 25% in publicly-financed healthcare centers, with significant variations between provinces (13%–28%) and centers [1].

In response to the rise in CS rates across Spain, the Ministry of Health released in 2008 a joint policy statement aimed at reducing unnecessary CS and promoting normal childbirth whenever possible [4]. However, these recommendations have remained quite general and decisions to opt for CS continue to be discretionary and often based on non-medical factors [5].

Although evidence on the effectiveness of various strategies to reduce cesarean rates is limited [6, 7], some studies that evaluated interventions targeting health care providers (obtaining second opinions, conducting audits with feedback, having peer reviews) suggest that such interventions are promising and have brought reductions in both cesarean rates [8–12] and mother-child morbidity [8].

In Spain, a multicenter program was implemented at the national level by the Ministry of Health, with leadership in the Balearic Islands. The aim of this program was to reduce the number of inappropriate cesarean sections in public hospitals [13]. In Andalusia, the program was introduced in 20 public hospitals. The steps taken in this program included training professionals in best practices, performing technical audits of cesarean delivery data and giving feedback to the professionals. The audit process allowed inappropriate CS practices to be identified and corrective actions to be defined [14].

The preliminary results of the program show a decrease in obstetric interventions, although rates vary considerably among hospitals [14]. Further analysis is needed to better understand which elements of the program have acted as barriers and which have acted as enablers to achieve the program goals, how the knowledge generated can best be applied, and how existing models can be transformed so as to improve childbirth practices and system performance.

Recent qualitative studies have explored the factors interacting in the childbirth care process, at the policy/management level, at the organizational level and in relation to the women giving birth [15–20]. These factors create a complex environment of barriers and enablers that could make the reduction of the cesarean rate more difficult [15]. However, no specific studies have been found on precisely how these factors intervene, particularly in a knowledge translation project intended to reduce the rate of cesareans.

The purpose of this study was to identify the factors that have been conducive to changing practices and reducing CS, as well as the challenges faced by professionals and hospitals in their efforts to implement the program for cesarean adequacy (PCA). The factors considered relate to policy/management, hospitals, practitioners and patients. This evaluation was carried out before the end of the program, to identify the need for adjustments and to promote optimal implementation.

## Methods

A qualitative exploratory study was performed in the region of Andalusia (Spain). Participating in the study were ob-gyns and nurse-midwives working at 14 hospitals where the program was being implemented. The study was performed 6 months after initiation of the program. The PCA is explained in detail in the protocol published elsewhere [14].

### Research technique

To gather information, in-depth individual interviews were used. The interview script was developed by the research team, using the dimensions influencing childbirth care identified by Chaillet [20] (Table 1).

**Table 1:** Dimensions explored in the interview script

Dimension	Aspects to discuss
Factors related to healthcare policy and management	• Local policies • Leadership • Organizational aspects • Economic incentives • Availability of equipment and personnel
Factors related to hospital characteristics	• Hospital policies • Service provision • Type of infrastructure (care level) • Culture in terms of care compensation (values, principals) • Care organization (primary and specialized care) • Training • Quality control and risk control • Communication mechanisms • Collaboration among departments
Factors related to the motivation and attitudes of healthcare professionals	• Medical-legal problems • Information and support provided to the women • Aptitude levels • Acceptance of guidelines • Strategies used to put the recommendations into practice
Factors related to the women giving birth and their families	• Motivations • Demands • Perceived needs

**Table 2:** Types of Hospitals in Spain’s National Healthcare System

Hospital category	Catchment area	Medical specialties
Regional Hospital	Entire region	All
Specialty Hospital	Provincial	Many
Local Hospital	Population living at distance of 1 h or less	Basic
Tertiary Hospital	Population living at distance of 30 min or less	Many

**Table 3:** Profiles of persons interviewed

Code	Professional profile	Type of Hospital	Sex	Supervises others	Cesarean rate pre-intervention period (%)
1	OB-GYN	Regional	Female	Yes	24,2
4		Regional	Male	Yes	22,2
3		High Resolution	Male		23,6
2		Local	Female		27
5		Local	Male	Yes	30,2
6		Local	Female		23,0
7		Regional	Female		26,2
8		Regional	Female		19,8
9		Specialty	Female		21,3
10		Regional	Female		25,9
11		Local	Female	Yes	23,7
12		Local	Male		15,7
13		Local	Female		19,8
14		Local	Male		19,0
15	Nurse-midwives	Regional	Female		24,2
18		Regional	Male	Yes	22,2
17		High Resolution	Male		23,6
16		Local	Female		27
19		Local	Female		30,2
20		Local	Male		23,0
21		Regional	Female		26,2
22		Regional	Female		19,8
23		Specialty	Male		21,3
24		Regional	Female		25,9
25		Local	Female	Yes	23,7
26		Local	Female		15,7
27		Local	Male		19,8
28		Local	Female		19,0

**Table 4:** Summary of program barriers and enablers related to healthcare policy and management and to hospital characteristics

Dimensions	Barriers and enablers	Dimens.	Barriers and enablers
Factors related to healthcare policy and management			
Policy and macro-management	Barriers: - Institutional policy has limited capacity to influence real clinical practice - Low commitment by the healthcare center to implement policy - Lack of investment by upper management levels - Distance between institutions and the day-to-day reality of healthcare centers - High degree of centralization of the healthcare system and little collaboration among centers Enablers - National plans and policies aimed at reducing cesarean rates	Organization of center and personnel management	Barriers: - The hierarchy of doctors and that of nurse-midwives is separate, with two different management lines - Rigid structure makes it difficult to establish incentives for good clinical practice and sanctions for poor clinical practice - The reorganization of competences between nurse-midwives and Ob-Gyns has created conflict - Departments besides obstetrics/gynecology are not involved in or even aware of project Enablers - Availability and disposition of anesthesiologists - Good coordination with Pediatrics and Emergency Departments
Factors related to hospital characteristics			
Characteristics of personnel and hospital	Barriers - Resistance to change shown by some professionals and the difficulty of “unlearning” the way things are usually done. - More years of professional practice perceived as a factor that heightens resistance to updating practices. Enablers - The close communication in small hospitals can introduce more elements that push personnel to update their practice.	Training of personnel	Barriers - Taking days off work for training is now more difficult than before - Personnel must assume cost of training Enablers - Training has allowed professionals to update their knowledge and skills for less interventionist deliveries, and it has also contributed to a change in the professionals’ mentality
Cooperation within the department and with Primary Care	Barriers - Nurse-midwives describe themselves as more inclined to non-intervention than the medical personnel but it is the latter who make the final decision about the delivery - Many primary care centers do not have nurse-midwives on staff Enablers - Efforts and initiatives to improve cooperation between nurse-midwives and Ob-Gyns - The information and guidance provided by nurse-midwives on staff at primary care centers	Leader-ship	Barriers - The PAC not being presented and explained to the staff - Hospitals without leadership or with recently-established leadership Enablers - Managers who are actively involved: motivating, raising awareness about the program, facilitating access to the necessary resources, providing supervision and evaluation - Managers who have capacity to negotiate, a good knowledge of the department and the staff, the ability to delegate, appropriate training, communication skills
Availability of human and material resources	Barriers - Absence of monitoring equipment - Obsolete delivery rooms with a medicalized appearance - Distance between delivery room and operating room - Shortage of nurse-midwives in deliveries without complications and shortage of medical personnel in deliveries with complications	Enablers - Having a pH meter available provides clinical and legal backing and facilitates adherence to the recommended time periods - The remodeled dilation-delivery units facilitate care circuits, making work more fluid

**Table 5:** Summary of program barriers and enablers related to the healthcare professionals and to the women and their families

Dimensions	Barriers and enablers	Dimensions	Barriers and enablers
Factors related to the motivation and attitudes of healthcare professionals			
Legal and medical pressure and professional prestige	Barriers - Professionals end up doing Defensive Medicine or conservative clinical practice; the specific nature of obstetrics and gynecology mean the pressure is higher in this specialty. - Doing a cesarean is considered a safe-conduct in the event of a legal dispute - The responsibility falls more on medical professionals than on midwives Enablers - Pressure due to professional prestige, and individual responsibility	Econ-omic incen-tives, compensation	Barriers - Absence of non-economic incentive system - Questions regarding validity of individual evaluations of personnel. Suspicion that the audits do not take into account the causative factors Enablers - The economic incentives are low and do not appear to be enablers - Satisfaction gleaned from doing a good job, recognition by the patients of the care provided - The audits, if they are positive, serve as positive reinforcement of an individual’s clinical practice and if they are negative they facilitate improvement.
Personal situation and clinical skill	Barriers: - Demotivation caused by changes related to the economic crisis and deterioration of working conditions. - Practices sometimes based on convenience for the professional	- Some obstetric practices are no longer used, such as external version, and know-how is being lost because they are no longer taught. Some instrumental practices are not as well known as before, while providers continue to use and have a good command of cesarean deliveries.	
Factors related to the women giving birth and their families			
Pressure on professionals	Barriers - Fear of pain - Bad experiences in past or of close friends/family. - Impatience in waiting for delivery to progress naturally - Pre-conceived ideas about the “ideal delivery”. Enablers - Improved information circuits for patients and their families	Trust in the professionals	Barriers - Reduced prestige of hospital - Distrust and fear of National Healthcare System and its professionals. Enablers - The main fear for conquering fears and prejudices is communication. - Communication and information exchange during entire gestation is fundamental: women in labor are less likely to assimilate new information.
Information	Barriers - Misinformation based on confusion and myths - Excess of information may overwhelm women - Contradictions in information from the private system (used for care during pregnancy) and the public system	Role	Barriers - Patients who have little decision-making capacity - Women who want to have “too much” control Enablers - Patients who are well-informed by midwife and gynecologist and do not have too many external influences - Patients who know that birth is physiological and natural and the less intervention the better

### Sample

Structured and purposeful sampling of ob-gyns and nurse-midwives was used. Criteria for inclusion was having at least one year of work experience at the hospital and having participated actively in the PCA. Two professionals in each centre were interviewed. To access the participants, the heads of the relevant departments were contacted, the initial contact was by e-mail invitation explaining the purpose of the study and requesting the names of two professionals (one ob-gyn and one nurse-midwife) who fulfilled the criteria. The sample of participants consisted of 28 persons with heterogeneous characteristics. The interviewee profiles and hospital characteristics can be found in Tables 2 and 3.

The average age of participants was 45 years old (range 37 to 55) with an average of 15.6 years of professional experience. Among the 28 participating individuals, 10 were men and all were from Spain.

### Information gathering procedure

The interviews were conducted between September and December of 2015 by two trained interviewers. Interviewers were not the researchers. Before the session, participants were informed of the study’s objectives, verbal consent to record the conversation was requested and consent to participate was obtained in writing.

### Information analysis

A cyclical, reflexive and flexible process for analysis was used, guided by the analytical approach put forward by Taylor & Bogdan [21], the phases of which (adapted for this work) are: 1) Familiarization and discovery: searching for themes and categories, examining the data in all possible ways, 2) Reduction and coding, 3) Analysis of data referring to the themes, ideas, and interpretations related to the objectives, 4) Verification and relativisation of data: Data is interpreted within the context in which it was gathered and bias control and triangulation of results is revised. As the interviews were conducted, they were transcribed with the software program SoundScriber. Then an initial reading of the documents took place, identifying the emerging ideas, coding the texts and identifying the meanings and the discourse in relation to the categories established. The software program NVivo 11 was used to organize the data. Finally, work began on the coded texts and field notes. To improve research quality, two researchers worked in collaboration until they arrived at a consensus on the initial findings. To do so they carried out second readings and analyses of the interviews [22].

## Results

### Characteristics of the sample

As shown in Table 3, the informants were 14 ob-gyns and 14 nurse-midwives, of whom 18 were women and 10 men. They came from 7 local hospitals, 5 regional hospitals, 1 specialty hospital and 1 high resolution specialty hospital.

In Tables 4 and 5 there is a summary of program barriers and enablers related to healthcare policy and management and to hospital characteristics and to the women and their families.

#### Factors related to healthcare policy and management

In general, the informants cited the lack of coherence between the healthcare policies and day-to-day activities. Although institutional actions like the PCA were viewed positively, however, such initiatives had limited influence and there was no real commitment to implementing the projects. In the view of the professionals, the budget allocated for these programs was insufficient.

- *It is always a paradox and the humanization [of childbirth] is a beautiful concept (…) but there is no real commitment by healthcare centers to require professionals, or units, to give a certain type of care, to act a certain way, to provide women with a certain type of access to information (Female, Ob-Gyn, Regional Hospital, Supervises others)*

- *In principle the hospital's values agree [with the policy] (…) theoretically the hospital works in favor of it all, right? But it's a different story when money has to be spent. That's where things get difficult (Female, Ob-Gyn, Local Hospital, Supervises others)*

Some informants pointed out that the professionals at the Andalusian Ministry of Health often did not take into account the day-to-day activity of a hospital.

- *Well, practice may be changing in the books they write, but real clinical practice is something else entirely. One thing is what they write, a few of them get together and write books and send a photocopy to the hospitals and another thing altogether is clinical practice (Male, Ob-Gyn, Regional Hospital, Supervises others)*

Another problem that acted as a barrier, was the atomized organization of the network of centers within the healthcare system, which was not conducive to communication and cooperation among hospitals. For example, the separation between the heads of the departments of nursing and gynecology is an obstacle to making messages consistent and to monitoring professional practice. Furthermore, those who occupy positions of responsibility lament the shortage of means with which to sanction poor clinical practice by the personnel under their supervision.

- *One of the biggest problems here is that we are restricted, (…) we cannot really reward a person who does good work nor can we punish a person who doesn't do such a good job. Yes, some objectives have been introduced to be able to appraise them but they are still subjective. I think we need more tools for correctly measuring the work we do, how we do it and why we do it (Female, Nurse-Midwife, Regional Hospital, Supervises others)*

The PCA called for the reorganization of some of the competencies of midwives and ob-gyns. This process has not been conflict-free and it has had an impact on the roles adopted and the responsibilities assumed, thereby affecting the relationships between professionals in the delivery rooms. This was not properly addressed at the management level; the competences were not determined in advance by the parties involved.

*It has been very difficult because we have clashed as groups (…), on the one hand professionals of my category did not want to adopt the ob-gyn's competences; and, on the other hand, because the other category was like “this has to be done because I am in charge and I say so” (…) And that causes friction (…). Attempts are being made but I think the planning was not good, because it is only when a problem arises that the different competences are specified (Female, Nurse-Midwife, Regional Hospital, Supervises others)*

#### Factors related to the hospital characteristics

Cooperation between care levels, between different hospital departments and within the gynecology/obstetrics department itself is considered a fundamental factor by the professionals interviewed. For example, the specialized information and training offered to women by midwives at the primary care level had positive results easily perceived by the professionals who attend deliveries.

*- I see a big difference when a woman comes from a primary care center that has a midwife on staff as opposed to one that does not have a midwife, because she hasn't been to prenatal classes, she hasn't drawn up a birth plan, she is not well informed (13. Female, Ob-Gyn, Regional Hospital)*

*- A woman who has not been to good pre-natal classes, or has not had visits with midwives at her primary care center, is a woman who basically only knows what her next-door neighbor says. Then we have to struggle against misinformation (21. Female, Nurse-Midwife, Regional Hospital)*

Similarly, the relationship between the gynecology/obstetrics department and the anesthesiology and pediatrics departments is considered an enabling factor. Particularly important is the relationship with the anesthesiology department, because this is where obstacles hindering the achievement of the PCA’s objectives have been identified in some hospitals.

- *To meet the program's goals, the main obstacle or one of the main obstacles are the anesthesiologists (…) I realize we have some very good anesthesiologists here but they also are overloaded with work: “Look” [some of them say] “instead of waiting around until 5 in the morning, let's just do it now and be finished earlier” (27. Male, Midwife, Local Hospital)*

As for cooperation within the gynecology/obstetrics department itself, some suggest there is good communication between professionals and a high degree of teamwork. However, the group of midwives describes itself as more in favor of vaginal birth, more in favor of non-intervention by the medical staff. The final decisions about delivery are made by medical staff and not all midwives feel they are able to follow the PCA’s recommendations. Differing visions of childbirth and also the respective roles and responsibilities of midwives and ob-gyns influence the professional relationship between them and when conflicts increase, the pressure on midwives also increases.

- *(…) midwives are, by default, more inclined to opt for fewer cesarean deliveries, that is, we prefer to wait until the last minute (…), and even give the woman a little extra time. And yes, it may be that we feel pressured by gynecologists, or anesthesiologists. They may have less patience when it comes to reducing the rate of cesarean deliveries (27. Male, Midwife, Local Hospital)*
- *High interventionism by ob-gyns during the process and the fact that they do not let midwives work as we would like to, (…) when we want to explore the woman every 2, 3, or 4 hours, we have the gynecologist looking over our shoulders saying “do an exploration, if her waters haven't broken, then break them, if you haven't administered oxytocin, give her some” (…) so the midwife feels this pressure (24. Female, Midwife, Regional Hospital)*

Some participants revealed that efforts are being made to increase cooperation between midwives and gynecologists and thus facilitate adherence to the PCA.

- *I think we have had some rough stages in terms of groups of professionals, with each one staking out its own place, and I think that's inevitable at times. But overall a lot of work is being done in this regard, with meetings for everyone, so that everyone has a say in any changes being made (10. Female, Ob-Gyn, Regional Hospital)*

A second group of factors identified at the hospital level is related to the professional’s perception of the **infrastructure and hospital resources**. For some professionals, it is a factor that facilitates the success of the program, while for others it is considered an obstacle.

Professionals who work in hospitals not equipped with advanced **fetal monitoring systems** view its absence as a barrier, something that makes it difficult to comply with the recommendations of the program. Uncertainty regarding fetal well-being makes it difficult to follow the recommended wait times before cesarean and the general discourse is that having the appropriate equipment, such as a pH meter, provides the staff with both professional and legal support, and this makes it easier to comply with the indicated wait times.

- *[What would most help is having…] a pH meter right when we need it, and not seeing later that most of the cesearans done for loss of fetal well-being are unnecessary but are performed because we don't have that device (3. Male, Ob-Gyn, High Resolution Specialty Hospital)*
- *For me it is hard to follow the recommendations because I do not have a pH meter in the delivery room. When I want to look at pH levels, I have to take the woman to the delivery room, extract a sample and send it to the analyzer, which is in the ICU. (...) this makes things difficult (18. Male, Ob-gyn, Regional Hospital, Supervises others)*

On a similar note, the general perception is that the remodeling of the childbirth units has brought improvements in the women’s comfort. The renovated facilities are more conducive to circuits of care, making the work more fluid and enhancing the possibilities of monitoring the birth. In this respect, the new dilation-delivery units are perceived as enablers of the PCA.

*-It is much more comfortable for the woman and for us as well (…). Imagine there are eight women dilating, having them all there is fantastic (…). I believe that a lot of progress has been made in the way care is provided, so that it is all more dynamic. Before not all the induction protocols were followed because there wasn't enough sPCAe (…) (8. Female, Ob-Gyn, Regional Hospital)*

Some informants warn that simply having the right infrastructure is not enough. This infrastructure must be accompanied by an appropriate number of professionals, for maximum benefit.

- *There is a limited number of midwives (…). The best thing would be to have one midwife for every woman in labor, but sometimes a midwife is attending more than one woman, with everything that such a situation implies (18. Male, Ob-Gyn, Regional Hospital, Supervises others)*
- *It depends on how much work there is, since we have four delivery rooms and two midwives on a shift (…). There are times when if you don't break the waters, even though [the recommended] 4 hours have not yet passed, you have to break the waters, because otherwise we aren't making progress, are we? (21. Female, Midwife, Regional Hospital)*

Training is an important element in the effectiveness of the PCA, because it updates the professionals’ knowledge and skills for a less-interventionist practice, but training is also a good tool for increasing awareness.

- *(…) it depends a lot on the professional attending the birth. There are professionals who have better training and have made an effort to adapt to the changing recommendations. For example, I was taught that episotomies should be done systematically and I have had to unlearn that (…) but there are a lot of people who have not jumped on that train and this means behavior by professionals varies a lot (24. Female, Midwife, Local Hospital)*

The leadership and involvement of Department Heads is a fundamental enabler. Some characteristics associated with good leadership have been identified: motivating personnel and helping them feel involved, raising staff awareness about the program, facilitating access to available resources, overseeing and evaluating the actions carried out, having the caPCAity to negotiate, a thorough knowledge of the department and the staff, willingness to delegate, being well trained and being able to communicate well with the team.

*-One very important enabler is the Department Head, they are the ones who can do the most to make something work. It's as clear as day (26. Female, Midwife, Local Hospital)*

- *Here we have been very lucky because [our Department Head] fights like a midwife. (…) So it has been pretty easy for us as doctors to introduce new things (Female, Ob-Gyn, Regional Hospital)*
- *To be a leader you have to show a caPCAity to negotiate, knowledge, the ability to implement what you are asking people to do (2. Male, Ob-Gyn, Regional Hospital, Supervises others)*
- *We have a Department Head who is totally obstetrical and he is one of the best trained persons in the whole issue of natural childbirth (…) he is always learning new things and telling us when we make a mistake, or when he makes a mistake (…). His enthusiasm is contagious and he is very demanding (12. Male, Ob-Gyn, High Resolution Hospital)*

#### Factors related to the motivation and attitudes of healthcare professionals

The most frequently mentioned and most intensely perceived barrier on the professional level is the medical-legal pressure on the professionals. The interviewees have witnessed a change in the vision of the persons under their care and this change has brought an increase in the claims filed for medical malpractice. The pressure is greater in obstetrics than in other specialties. The pressure exerted by women and their families, mainly on doctors, results in the professionals sometimes finding themselves “forced” to make decisions not based on clinical indications.

- *We professionals are demoralized by all the suits being filed against us. We are still very vulnerable in that area, because the patient is not always right (…). And to prove it you have had to go to court, you have had to stand before a judge (Female, Midwife, Regional Hospital).*
- *(…) there is pressure from society, there is a lack of protection from the institutions when a problem arises and our society is increasingly litigious, people always have to have a positive outcome (Male, Ob-gyn, Regional Hospital, Supervises others)*

One of the consequences of this medical-legal pressure is what is known in the discourses as *defensive medicine*. In the case of attending births this translates into more interventions. Not only because the cesarean is often perceived by providers and patients as safer, but because performing a cesarean is a form of protection should the provider be taken to court.

- *Whether we like it or not, we end up practicing a bit of defensive medicine, you see? I think that is the most important factor, we are afraid of the repercussions. The result is that the recommendations are not always followed (Female, Ob-Gyn, Regional Hospital)*
- *The only thing that releases the gynecologist when someone files a law suit later is “Look, I am going to do a cesarean delivery on time” and “Poor thing, the baby didn't come out right but at least I did the cesarean” (Male, Ob-Gyn, Local Hospital)*

The changes that the economic downturn has caused in the healthcare system, such as deteriorating working conditions, have led to demotivation among healthcare providers.

- *We feel demotivated and this affects not just the reduction of c-section rates, which may be more general, but also the PCA itself. The cutbacks in the hospital have really been felt… (Male, Midwife, Local Hospital)*

There is also a general perception that some obstetric practices are no longer used, such as external versions, and that some clinical skills and abilities are being lost because they are no longer taught. Some instrumental practices are not as well known as before, while providers continue to use and have a good command of cesarean deliveries.

- *Other times there is a fear of solving a dystocia problem with an instrument because the professional doesn't know how to use it (…). Ob-gyns from 30 years ago did breach deliveries and used forceps frequently and were very skilled. Now I'm seeing the opposite, that certain groups of doctors do not know how to solve dystocias vaginally (…) so the cesarean is viewed as the simpler solution (Female, Midwife, Local Hospital)*

Economic compensation does not appear to be a factor influencing effective implementation of the program. The rate of cesarean deliveries tends to be one of the objectives set by the healthcare centers and it is linked to an economic supplement for professionals who meet the objectives. However, this compensation is viewed as a residual element, not a great deal of money considering the effort that must be made to receive it.

- *The objectives affect us economically but I don't think anybody would do a cesarean thinking about the supplement and the objectives (…). I don't think any of us have that in mind when we decide whether or not to do a cesarean (Female, Ob-Gyn, Local Hospital)*

However, other types of rewards more related to the sense of satisfaction brought by doing good work, to receiving recognition from patients for the care provided during delivery, and to having easy access to the resources necessary for your work, are described as factors that have an influence on adherence to the PCA.

- *I think that what most stimulates the staff is that people trust you, that the mothers are happy, that you are given the resources you need to do a good job, that any shortage of materials is remedied (…) (Female, Midwife, Local Hospital)*

In general, audits of healthcare provision are perceived as useful. Individual evaluations, when they are positive, are a reinforcement of individual clinical practice and this affects the provider’s motivation to keep working to achieve good results.

- *Analyzing the data afterwards is definitely a point in our favor. And there are things about which we can say “this could have been better” or “there is a conduct in this type of patient that we can change”. When the results are analyzed afterwards, it is always a learning experience, you always get something out of it (Female, Ob-Gyn, Local Hospital)*

#### Factors related to the women giving birth and their families

Another group of factors affecting efforts to reduce cesarean deliveries is related to the characteristics of and the demands made by women and those who accompany them during labor. The informants agree that how much and what kind of information the women have is important. There is generalized concern about either a lack of information regarding the delivery or the presence of colloquial information, transmitted by word of mouth, which sometimes means that women have erroneous, partial and subjective information that is plagued by myths and confusion.

- *I spend a lot of time explaining things, alleviating fears, offering them the tools that are available and encouraging them to decide how we should proceed. But most of the women here just follow along, they are not well-informed (…). Sometimes they ask you something and you have to say to yourself: “whoa, it looks like we're starting from zero” and it's a real shame (Male, Midwife, Local Hospital)*
- *Mainly it is: “I heard that…, my cousin told me that… and she had a cesarean because…and so on and and so forth”. So, knowledge passed around “mouth to mouth” limits us a great deal because the women come in with a concept in mind, with a pre-conception already formed and you have slowly undo it (Female, Ob-Gyn, Regional Hospital)*

Another barrier perceived is excessive information or information that comes from informal sources, such as Internet or magazines. This can generate mistrust and apprehension by patients towards the public healthcare system. This situation is worse when the woman has received maternity care twice over, within the public system and, at the same time, through the private system, because sometimes the information the women receive does not coincide. In addition, the profile of well-informed women is perceived by some groups of informants as an enabling factor for implementation of the PCA while in other groups it is perceived as a negative factor, since this profile contains elements that can contribute to patient-provider clashes.

- *Since they have more information, but often misinformation, they think that a cesarean has a lot of advantages or poses fewer problems for them, when in truth it is the opposite (Female, Midwife, Local Hospital)*
- *The patient profile that most facilitates [vaginal delivery/adherence to the PCA recommendations] is the woman who is well informed and motivated with regard to her pregnancy, to her delivery, and who does not have too many external influences. The best thing is for the women to be well informed by their midwife, their gynecologist and for them to be aware that childbirth is a natural process and that the less intervention by us the better. That is the ideal patient (Male, Ob-Gyn, High Resolution Hospital)*

Good information, viewing childbirth as a biological phenomenon, having a predisposition to a vaginal delivery and also a certain level of empowerment come together and are found in a new profile of women giving birth, a profile that one ob-gyn has called “biological” patients. Some discourses point out the necessity of women having sufficient information to make their own decisions, since their collaboration is considered to be closely related to obstetric success. The fact that they are involved in the process and feel empowered is interpreted as a positive element.

- *The ones that we call “biological” patients, these patients facilitate vaginal deliveries. (...). They are the ones who have decided that they want a low-intervention birth, they don't want an epidural, these women facilitate vaginal deliveries, of course. Female, Ob-Gyn, Specialty Hospital)*

Women’s lack of empowerment during childbirth also has effects on the monitoring of clinical practice, that is, on the possibility that women will demand improvements in the care provided and advocate the elimination of poor clinical practices. However, there are providers who link the determination of some women who want a certain type of birth, who want to have “too much control”, with a conflictive profile that makes it difficult to provide proper care. On the other hand, the fact that a woman arrives at the hospital asking for a cesarean, in the absence of medical indications for it, not only means greater pressure on the personnel attending the delivery; it may also interferes with the birth itself, contributing to the appearance of obstacles that can affect the natural course of labor.

- *There are women who do not know what to decide, they don't know what they want and that is very bad (…) it's a very important moment in your life, with major implications for your future, for the health of your child, for your health, for your happiness and satisfaction regarding the birth (…). I encourage all women to take the reins of their deliveries (Female, Ob-gyn, Regional Hospital, Supervises others).*
- *A woman who arrives at the delivery room not wanting a vaginal delivery; if she wants a cesarean but we do not agree to it, that woman has a high chance of ending up needing a cesarean. (Male, Ob-Gyn, Regional Hospital, Supervises others)*

The main tool that healthcare personnel can use to conquer the fears and prejudices that patients have about vaginal delivery is communication. However, women in labor are less likely to assimilate new information. That is why enhancing communication with the patient from the outset, strengthening the network of pre-natal education and deepening information throughout the pregnancy is often put forward as a solution.

- *Simply providing the right information, that and the caregiver's aptitude and attitude are essential and have positive repercussions on how everything goes (…) and of course not providing information has the opposite effect (Male, Ob-Gyn, High Resolution Hospital)*
- *If their primary healthcare center has a midwife on staff, there is a big difference, yes, because the women know what a birth plan is, they know what the epidural is like and when and how it is administered, they know that we have a birthing tub, that there can be a low-intervention delivery…. The rest think that coming to give birth is whatever pops up. And the women who get information from the Internet are misinformed most of the time (Female, Midwife, Regional Hospital)*

There is a widespread idea that at times the pressure by families causes decisions to be made without sufficient justification. The pressure can be so intense that some informants speak of aggression and threats.

- *And the pressure by the family, and by the woman, (…) that pressure hammers away at you, and it even makes the gynecologist say “I don't really know because I cannot fight against the woman, her husband, her mother-in-law and her aunt who told her that she knows someone who swallowed liquid and I don't know what else” (Male, Midwife, Specialty Hospital)*
- *It is hard to get rid of the pre-existing cultural and social thinking, and transmit to them that sometimes they just have to wait a little longer. They say “somewhere else they would have done the cesarean half an hour ago” or “if something happens to my baby I'll come looking for you” or “a friend of mind did such and such and I want to do it the same way” (Male, Ob-Gyn, Local Hospital)*

## Discussion

This study has identified different factors that have facilitated or hindered the effectiveness of the national program to reduce the rate of cesarean deliveries in Andalusia’s public hospitals. In-depth interviews were found to be a good technique for learning the opinions and perceptions of the healthcare professionals involved in the program. This dialogue has led to a better understanding of aspects of the problem that remain latent and that emerge in the day-to-day activities of healthcare professionals attending births in public hospitals.

There are limitations to this study. This analysis may not reflect the situation in other regions, which could have different problems with access, provider relationships, or medical-legal climate. Qualitative research cannot be generalized [23], but to a theory of the phenomenon being studied, in our case, programs to reduce cesarean rates.

The PCA cesarean reduction program is based on a process of audit and feedback. Audit and feedback is a common implementation intervention that has been used in a variety of clinical contexts and evaluated in three Cochrane reviews and updates over the past 30 years [24–27]. In the field of cesarean reduction, it is a very promising methodology, although results to date have been modest [7, 8]. The results of this study can contribute to the design of more effective interventions based on overcoming obstacles, highlighting enabling factors and making an effort to (re)define the boundaries between research and practice.

In Spain, a number of transformations are taking place within the National Healthcare System and in the external factors that influence it, which in turn have an effect on the PCA. Among the healthcare policy changes that affect obstetrics departments, the reorientation of clinical practice toward the humanization of delivery has been especially significant and has brought important changes on different levels [26]. The strategy set by Spain’s healthcare system is to work towards establishing consensus regarding the naturalization of childbirth and to equip healthcare centers with the human and material resources necessary to favor vaginal deliveries.

The new focus on the humanization of perinatal care and the effort to reduce the rate of cesarean deliveries are part of the demand for a less interventionist clinical practice, which would imply a greater role for midwives and a change in the relationship between obgyns and midwives, along with a new distribution of competencies [28, 29]. This new context of relations is not free of conflict, as this study shows. The change in professional practice sought by the PCA project is one of the fruits of these developments. Two recently released, unrelated reports serve as stark reminders of how challenging it has been to change professional behavior in a sustained and purposeful way [30–32].

The new framework also entails incorporating shared decision-making into the birth process, giving greater agency to the women, their decisions and their needs during pregnancy, delivery and puerperium. Légaré [33] notes that shared decision-making involves several essential elements. First, providers and patients must recognize that a decision is required. Next, they must have at their disposal, and understand, the best available evidence. Finally, they must incorporate the patient’s preferences into treatment decisions.

There are elements in this study that must be viewed as core issues. For example, midwives perceived themselves as more inclined than doctors, to adhere to the PCA and it points to the medical staff as being less inclined to intervention. The discourses underlying these opinions reflect a different attribution of responsibilities and recognition of the role of midwives. While some informants were aware of the importance of midwives in reaching the project’s goals, others underline that the decision to perform a cesarean is based on the doctor’s judgment alone. Some studies showed that the influence of the model of maternity care concerns (more humanised vs. more technology focused) not only the obstetric results but also the level of mother’s satisfaction. Higher mother’s satisfaction was found in a more humanised models of care compared with the biomedical one [34]. Other authors have also demonstrated that high levels of interventions in normal birth can lead to the dissatisfaction of women and their families [35]. The discourses formulated from feminist viewpoints or more egalitarian positions are inclined to defend greater empowerment for pregnant women because it will lead to monitoring of the delivery process and clinical practice. These discourses contain more criticism of the patriarchal and medicalized vision of childbirth and they highlight the characteristics of “biological patients” as being positive elements. More paternalistic discourses defend information being provided to pregnant women because it can reduce conflict and contribute to the acceptance of medical criteria, they consider “biological patients” to be conflictive and they are less critical of the caregiving activity and clinical practice of the personnel. Ensuring that women can take active part in their pregnancies and deliveries, can experience a less-medicalized birth in a setting of mutual listening and active collaboration among all parties, is to broaden the right of women to inclusive health and must be treated as a priority [1].

In future research, it would be interesting to examine the opinions and expectations of women giving birth. It would also be valuable to have a better understanding of the hierarchical relationships among different professionals working in delivery rooms, through the methodologies based, for example, on participant observation [36–38].

## Conclusions

- Barriers to knowledge translation of interventions to reduce cesarean sections are related to policy and management, organization, characteristics of the personnel and hospital, available resources, medical-legal pressure and the pregnant women and their families.
- Among the enablers to the reduction of caesarians, the most significant factors are good coordination with the pediatrics and emergency departments, the updating of professional skills for a less interventionist professional practice, and, for the women, awareness of the circuits of information for patients and families and trust in the professionals.
- The results of this study can be used to improve the design of interventions seeking more effective knowledge translation based on overcoming obstacles, reinforcing enabling factors, (re)defining the boundaries between research and practice.

## Abbreviations

CPGs: Clinical practice guidelines
CS: Caesarean section
QICS: Quality improvement for CS programs
